# Lactate dehydrogenase-elevating virus enhances natural killer cell-mediated immunosurveillance of mouse mesothelioma development

**DOI:** 10.1186/s13027-020-00288-6

**Published:** 2020-05-07

**Authors:** Mohamed F. Mandour, Pyone Pyone Soe, Catherine Uyttenhove, Jacques Van Snick, Etienne Marbaix, Jean-Paul Coutelier

**Affiliations:** 1 Unit of Experimental Medicine, de Duve Institute, Université Catholique de Louvain, SSS/DDUV – ICP, Av. Hippocrate 75, bte B1.75.02, 1200 Brussels, Belgium; 2 Department of Clinical Pathology, Faculty of Medicine, Suez Canal University, Ismailia, Egypt; 3 Department of Pathology, University of Medicine, Yangon, Myanmar; 4 Ludwig Institute, de Duve Institute, Université Catholique de Louvain, 1200 Brussels, Belgium; 5 Unit of Cell Biology, Université Catholique de Louvain, 1200 Brussels, Belgium

**Keywords:** Cancer immunosurveillance, Gamma-interferon, Mesothelioma, Natural killer cell, Virus

## Abstract

**Background:**

Viral infections can reduce early cancer development through enhancement of cancer immunosurveillance. This study was performed to analyse this effect of viral infection in a mouse model of solid tumor.

**Methods:**

The experimental model used was the effect of BALB/c mouse infection by lactate dehydrogenase-elevating virus on AB1 mesothelioma cancer development.

**Results:**

Acute infection with lactate dehydrogenase-elevating virus strongly reduced in vivo early AB1 mesothelioma growth and death resulting from cancer development. This effect was not due to a direct cytolytic effect of the virus on AB1 cells, but to an in vivo activation of natural killer cells. Gamma-interferon production rather than cytotoxic activity against AB1 cells mediated this protective effect. This gamma-interferon production by natural killer cells was dependent on interleukin-12 production.

**Conclusions:**

Together with other reported effects of infectious agents on cancer development, this observation may support the hypothesis that enhancement of innate immunosurveillance against tumors may result from infection with common infectious agents through modulation of the host immune microenvironment.

## Background

Although cancer remains a frequent and deadly disease, immunosurveillance prevents the development of most tumors in normal individuals. A large part of this immunosurveillance of cancer development is mediated by innate cells, especially by Natural Killer (NK) cells that have been shown to elicit very rapid anti-tumor responses through recognition of tumor-specific ligands by activating receptors, leading to elimination of cancers in their early phases of development [1, 2]. Both gamma-interferon (IFN-γ) and perforin participate to this NK-cell mediated immunosurveillance in normal animals, independently from concomitant infections [3–6].

Infection with many viruses induces a strong modulation of the immune system towards the proinflammatory Th1 pathway, leading to antibody isotype bias, a decrease in Th2 responses, production of proinflammatory cytokines and activation of cells with cytolytic activity such as NK cells and cytolytic T lymphocytes. Lactate dehydrogenase-elevating virus (LDV) is a common mouse arterivirus that triggers such a modulation of the host immune microenvironment [7]. In addition to other modulations of the immune system, LDV infection is followed by a strong activation of NK cells, that leads to high, but transient IFN-γ secretion and cytolytic activity, without alteration of viral replication [8]. Although the mechanisms responsible for NK cell activation after LDV infection have not been completely determined, they could include secretion of cytokines such as IL-12, IL-15, IL-18 and/or type I IFNs [7, 9, 10]. Through modulation of the host immune microenvironment, LDV has dramatic consequences on diseases that develop independently from, but simultaneously with the infection, with exacerbation of pathologies mediated by effector functions of cells such as macrophages that are activated by the infection [7]. However, in some circumstances, the same modulation of the immune microenvironment may result in an improvement of concomitant diseases [7].

Viral infections are also known to modulate cancer development, usually through direct interaction with cells. When this interaction results in cell transformation, viruses such as Epstein-Barr virus, hepatitis virus or papilloma viruses trigger cancer. In contrast, viruses that lytically infect cancer cells induce tumor regression. Moreover, alteration of immune microenvironment by viruses may also lead either to enhancement or suppression of cancer development [11–14].

Mesothelioma is a cancer with poor prognosis and rising incidence, often linked to exposure to asbestos. When appropriately activated, NK cells may provide help for systemic anti-mesothelioma immunity and for long term effector and memory responses [15]. In the mouse, treatment with TLR7 agonist retards the growth of AB1 cells, a close mouse model of mesothelioma, through mechanisms involving both NK cells and CD8+ T cells [16]. Moreover, interleukin (IL)-12 administration prevents the growth of the same tumor although it is not clear whether this effect depends on NK cells [17].

Therefore, we analysed in this work whether the modulation of the immune microenvironment that follows LDV infection may enhance immunosurveillance against AB1 and prevent its early development, and through which mechanisms. Our results indicate that acute LDV infection prevents mesothelioma growth through IL-12-dependent IFN-γ secretion by activated NK cells.

## Methods

### Animals

BALB/c and BALB/cAnNRj-Foxn1 nu/nu female mice were bred at the Ludwig Institute for Cancer Research by Dr. G. Warnier or were obtained from Janvier Labs and used at the age of 7–10 weeks. The project was approved by Comité d’Ethique facultaire pour l’Expérimentation Animale - Secteur des Sciences de la Santé - Université catholique de Louvain (ref. 2014/UCL/MD/008). Some of the mice had to be euthanized for ethical reasons.

### Virus

Infection was performed by i.p. injection of approximately 2 × 10^7^ ID_50_ LDV (Riley strain; ATCC, Manassas, VA) in 500 μl saline, as described previously [18]. This procedure was found to lead to infection of all injected animals.

### Tumor cells

AB1, a mouse mesothelioma cell line derived from mouse lung [19] was obtained from Sigma Aldrich (Public Health England, European Collection of Authenticated Cell Cultures [ECACC] General Cell Collection, Catalogue number 10092305) and maintained in RPMI 1640 medium containing 25 mM HEPES, 5% Fetal bovine serum (FBS, Gibco, Life technologies, Grand Isle, NY), 50 U/ml Penicillin G and 50 μg/ml streptomycin (Gibco, Life technologies), 2 mM L-Glutamine (Gibco, Life technologies). Exponentially growing cells were collected by brief trypsinization, washed twice with Phosphate buffered saline (PBS) and injected i.p. at a dose of 0.5–1 × 10^6^ cells in 500 μl PBS. Living cells were counted using Trypan blue staining.

P815 is a mouse mastocytoma cell line [20]. YAC-1 is a mouse lymphoma cell line used as target cell in NK cell cytolytic assays [21].

### Histopathology

The intestine, mesentery, liver and pancreas were fixed in 4% paraformaldehyde overnight. To evaluate tumor formation, the intestine with mesentery was cut into sections of 1 cm and tissues were included into paraffin blocks. Six micrometer thick transverse sections were cut with a HM 355S Automatic Microtome (Thermo scientific) and stained with hematoxylin and eosin. Slides were digitalized using a SCN400 slide scanner (Leica).

### Antibodies and NK cell depletion

Anti-asialoganglioside-GM1 (ASGM1) polyclonal antibody from immunized rabbit was used following a protocol shown previously to successfully deplete NK cells and to suppress their function [8, 22]. In vivo NK cell depletion was achieved by i.p. injection of 8 mg IgG in 500 μl saline 2 days before AB1 tumor cells administration, followed by injection of 8 mg IgG in 300 μl saline on the day of tumor cell challenge.

F3 rat anti-mouse IFN-γ monoclonal antibody (mAb) [23], purified with protein G-sepharose beads, was injected i.p. into mice at a dose of 500 μg 1 day before and 5 days after LDV infection.

MM12A1.6 (formaly named MMP35A1.6) mouse IgG2a anti-IL12 mAb [24] was injected i.p. into mice at a dose of 500 μg 1 day before and 7 days after LDV infection. C1407C3 mouse IgG2a control mAb was injected at the same times and doses.

### Cytotoxicity assay

NK cells were sorted from spleens of control and LDV infected mice using DX5 Microbeads (Miltenyi Biotec, Germany) according to the manufacturer’s instructions. 7-AAD/CFSE Cell- Mediated Cytotoxicity Assay Kit (Abcam, Cambridge, UK) was used to assess by flow cytometry NK cell cytotoxicity against AB1 tumor cells and Yac-1 mouse lymphoma cells. Background of target cells without effector cells was subtracted.

### Cell proliferation assay

AB1 cells were cultured in triplicates (5 × 10^3^ cells/well) in 96 well plates. Mouse IFN-γ (Biolegend, San Diego, CA) was added in serial concentrations (0.3, 0.9, 1.2, 5 and 10 U/ml) in 150 μl of medium. After 3 days, cell proliferation assay WST reagent (Roche) was added for 3 h to the culture medium according to the manufacturer’s instructions. The absorbance was measured at 480 nm.

### Flow cytometry

Flow cytometry analysis of IFN-γ-producing cells was carried out using BD-FACSVerse machine (Becton Dickinson, Franklin Lakes, NJ). Single-cell suspensions were prepared from spleens. Cells were first incubated for 4 h at 37 °C with 10 μg/mL monensin (Biolegend, Cat# 420701). γ-block was done using purified anti-mouse CD16/32 antibody (Biolegend, Cat# 101301). NK cells were labeled by surface staining with 1.0 mg APC-labeled anti-mouse CD49b mAb (DX5; Biolegend, Cat# 108909) per 10^6^ cells. For intracellular labeling of IFN-γ, cells were fixed and permeabilized using Cyto-Fast™ Fix/Perm Buffer Set (Biolegend, Cat# 426803) followed by staining with PE-labeled anti–IFN-γ mAb (XMG1.2; Biolegend, Cat# 505807). Data were analyzed by using FlowJo Software 9.8.1 (Tree Star, Ashland, OR).

### Statistical analysis

Results are expressed as means ± standard error of mean (SEM). When appropriate, one-way or two-way ANOVA with Bonferroni tests were performed using Prism 6 software (GraphPad Prism, La Jolla, CA, USA). Survival curves were analysed using Log-rank (Mantel-Cox) test.

## Results

### Prevention of mesothelioma early development after LDV infection

**Fig. 1:**
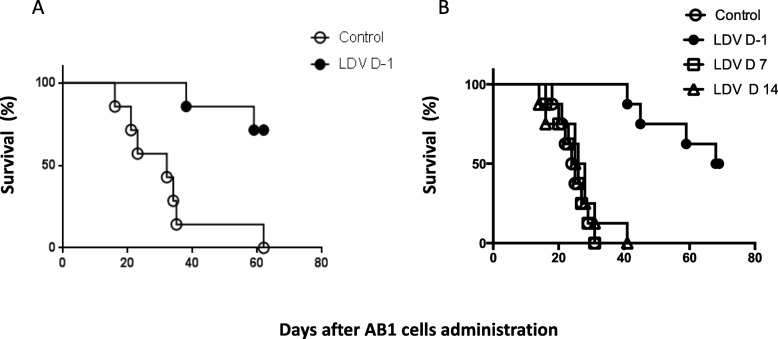
Preventive effect of LDV infection on early AB1 development. A. Survival of groups of 8 BALB/c mice either uninfected (open symbols) or infected with LDV one day before tumor administration (closed symbols), was monitored daily after i.p. administration of AB1 cells. B. Survival of groups of 8 BALB/c mice either uninfected (open circles) or infected with LDV one day before (closed circles), one week after (squares) or two weeks after (triangles) tumor administration, was monitored daily after i.p. administration of AB1 cells

**Fig. 2:**
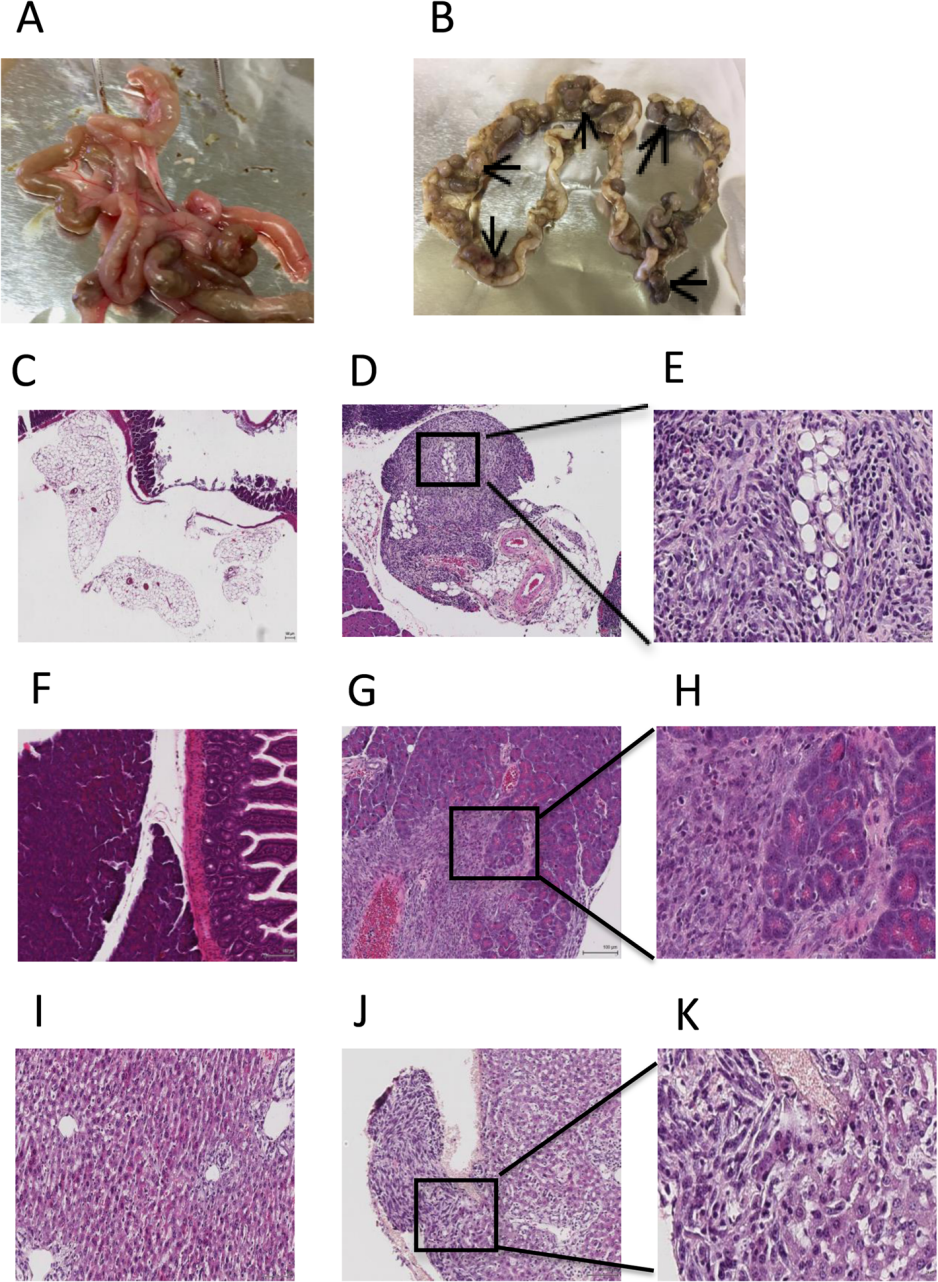
Histology of mesothelioma development. Analysis was performed 21 days after injection of 0.5–1 × 10 ^6^ AB1 cells in 500 μl PBS in control mice and in animals infected with LDV one day before (five mice per group). **a** intestine of LDV infected mouse; **b** intestine of uninfected mouse with small tumour nodules (arrow) attached to the mesentery; **c** mesentery and intestine of LDV infected mouse (no tumour) (4x); **d**, **e** Peritoneal mesothelioma in mesentery of an uninfected mouse (magnifications of 4x and 40x, respectively); **f** pancreas of LDV infected mouse (10x); **g**, **h** tumor cells infiltrating the pancreas in an uninfected mouse (magnifications of 10x and 40x, respectively); **i** liver of LDV infected mouse (10x); **j**, **k** tumor cells infiltrating the liver in an uninfected mouse (magnifications of 10x and 40x, respectively)

To determine the preventive effect of a viral infection on early mesothelioma growth, a low dose of AB1 cells was injected i.p. into mice one day after mock or LDV infection. Uninfected mice quickly started to develop tumors and most were dead 40 days after tumor inoculation _b_. In contrast, tumor development was prevented in LDV-infected animals and a majority of them were still alive after two months (Fig. 1a, p = 0.0011). A similar protective effect of LDV infection was obtained in 5 independent experiments. Histology analysis performed 21 days after tumor cell inoculation revealed microscopic tumor cell infiltration in the mesentery of five out of five uninfected animals, with tumoral nodules in one of them (Fig. 2b, d, e). Liver and pancreatic infiltration by tumoral cells was also noted in a few mice of that group (Fig. 2g, h, j, k). In contrast, no tumor lesions were found in LDV infected group, neither by macroscopic nor by microscopic examination (Fig. 2a, c, f, i). Tumor infiltration in the mesentery was also found in a majority of uninfected animals (3 out of 4 mice), but not in LDV-infected animals, in a second independent experiment.

In experiments where infection occurred one or two weeks after cancer cell administration, when the tumor had time to implant, the protective effect of LDV was no longer observed (Fig. 1b, representative of 2 independent experiments).

This impairment of AB1 tumor growth was not due to a direct cytopathic effect of LDV. Indeed, in vitro AB1 cell replication was not affected by the addition of LDV in the culture (Table 1, representative of 3 independent experiments).

**Table 1:** AB1 growth in the presence of LDV

Culture time^a^	cell count (×105)^b^	
(days)	without LDV	with LDV
0	1	1
2	2.8 ± 5.13	3.4 ± 1.73
4	9.4 ± 1.70	8.8 ± 1.25
7	11.7 ± 2.22	10.8 ± 1.36

^a^ 100,000 AB1 cells were cultured in 0.5 ml medium with or without 2 × 10^7^ ID_50_ of LDV. Cells were counted at different times after culture initiation

^b^ Cells were counted in triplicates, results shown as means ± SEM

### Role of NK cells in LDV-mediated prevention of mesothelioma growth

**Fig. 3:**
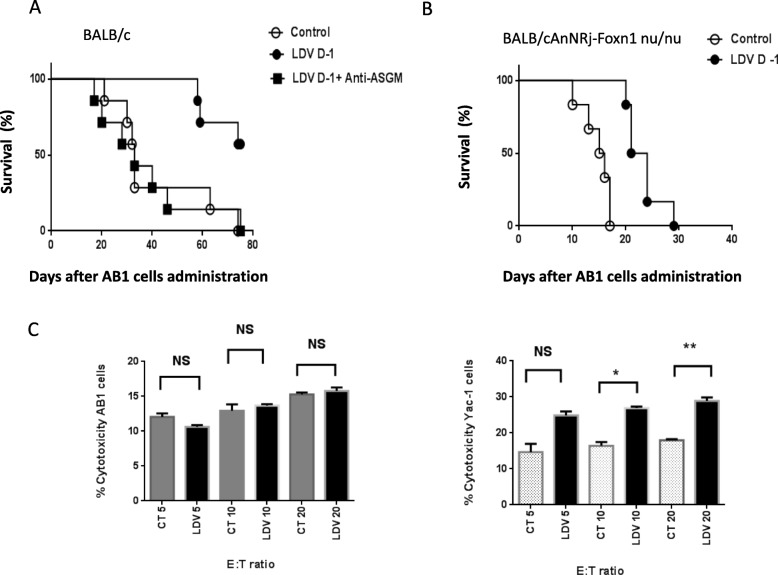
Role of NK cells and T lymphocytes in LDV-mediated protection against AB1 growth. **a** Survival of groups of 7 BALB/c mice either uninfected (open circles) or infected with LDV one day before tumor administration, with (closed squares) or without (closed circles) anti-ASGM1 treatment, was monitored daily after i.p. administration of AB1 cells. **b** Survival of groups of 6 BALB/cAnNRj-Foxn1 nu/nu mice either uninfected (open circles) or infected with LDV one day before tumor administration (closed circles) was monitored daily after i.p. administration of AB1 cells. **c** NK cell cytotoxic activity. Cytolysis of CFSE-labeled AB1 or Yac-1 cells (2.5 × 10^4^ cells/ml) was analysed by flow cytometry after 4 h incubation with serial ratios (E:T: effector/target cell ratio) of purified NK cells from control (grey bars) or LDV-infected (black bars) mice. Results are expressed as % of lysed target cells, mean ± SEM for groups of 3 mice. (*p < 0.05; **p **< 0.01)**

Since NK cells have been suspected to play a significant role in anti-mesothelioma immune responses, their involvement in the prevention of early mesothelioma growth after LDV infection was tested by treatment with a depleting anti-ASGM1 antibody. Such a treatment has been shown previously to efficiently deplete NK cells and therefore to suppress NK cell-mediated functions in LDV infected mice [8]. Anti-ASGM1 antibody administration resulted in a nearly complete suppression of LDV protective effect (shown in Fig. 3a for one representative experiment representative of two; difference with and without treatment: *p* = 0.0021). Because NK cells have been shown to provide help in systemic anti-mesothelioma responses [15], a possible involvement of T lymphocytes in the protective effect of LDV infection was tested in BALB/cAnNRj-Foxn1 *nu/nu* mice that are deprived of T cells. Control uninfected BALB/cAnNRj-Foxn1 *nu/nu* mice died much faster from mesothelioma than normal BALB/c animals, which indicates an important role of T lymphocytes in protection against this tumor (Fig. 3a and b). However, LDV infection still delayed death of these BALB/cAnNRj-Foxn1 *nu/nu* animals by approximately ten days (Fig. 3b, p = 0.0008). This suggested that T lymphocytes were involved in the overall control of tumor development, but that NK cells were required for the added protection conferred by infection. Such a relative protection of *nu/nu* mice was found in two independent experiments.

NK cells may exert anti-tumor activity through cytotoxicity or cytokine production. Although not with a significant difference for every E/T ratio, LDV infection enhanced NK cell cytotoxic activity against the classical Yac-1 target cells, as reported previously [8] (Fig. 3c). In contrast, the ability of NK cells to lyse AB1 cells was not as high and no difference was observed between NK cells from control and LDV-infected mice (Fig. 3c, observed in two independent experiments), suggesting that LDV protective effect against mesothelioma growth was not mediated by an enhanced cytolytic activity.

**Fig. 4:**
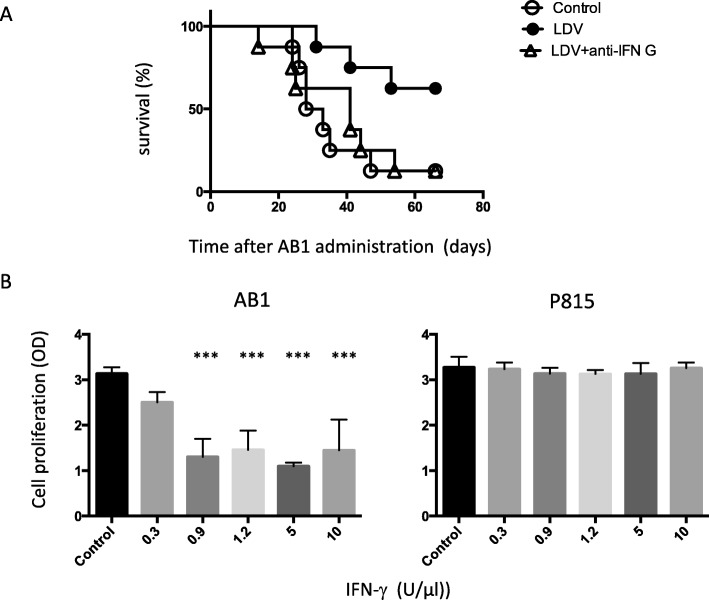
Role of IFN-γ in LDV-mediated protection against AB1 growth. **a** Survival of groups of 8 BALB/c mice either uninfected (open circles) or infected with LDV one day before tumor administration, without (closed circles) or with (open triangles) anti-IFN-γ treatment, was monitored daily after i.p. administration of AB1 cells. **b** Proliferation of AB1 and P815 cells was measured after 3 days of culture in the presence of serial IFN-γ doses. Results for triplicate measurement are shown as means ± SEM. ***: significant differences when compared to cultures without IFN-γ (*p* < 0.001)

Because NK cell activation after LDV infection results in high IFN-γ secretion [8], we analysed the role of this cytokine in virally-induced prevention of early mesothelioma development by treating infected mice with the neutralizing F3 anti-IFN-γ mAb. IFN-γ neutralization resulted in a suppression of LDV-induced preventive effect as complete as NK cell depletion (Fig. 4a, p = 0.036, representative of two experiments).

We then tested the sensitivity of AB1 cells to IFN-γ. As shown in Fig. 4b, addition of 0.9 U/ml IFN-γ to AB1 cell cultures strongly reduced their proliferation. In contrast the same treatment had no effect on P815 cells, a mastocytoma cell line on which LDV infection has been reported to have no protective effect [13]. This sensitivity of AB1 cells to IFN-γ was found in two independent experiments.

### Role of IL-12 in LDV-mediated prevention of mesothelioma growth

**Fig. 5:**
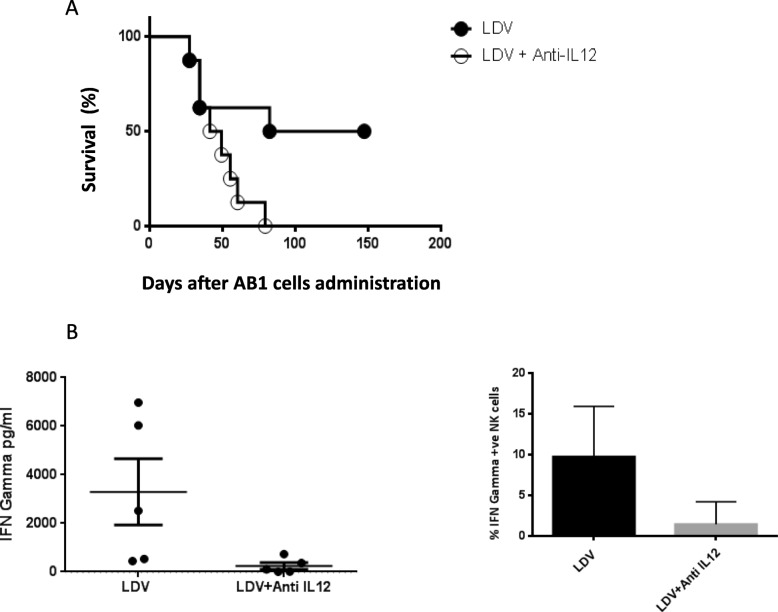
Role of IL-12 in LDV-mediated protection against AB1 growth. **a** Survival of groups of 8 LDV-infected BALB/c mice either without (open circles) or with anti-IL-12 mAb treatment (closed circles) was monitored daily after i.p. administration of AB1 cells. **b** IFN-γ was measured by ELISA in groups of 5 BALB/C mice 18 h after LDV infection with and without anti-IL-12 mAb treatment (500 μg 24 h before infection). **c** Flow cytometry analysis of IFN-γ–producing spleen NK cells 18 h after LDV infection with and without anti-IL-12 mAb treatment (same animals as in B). Results are shown as means ± SEM

IL-12 is known to be usually required for virally-induced IFN-γ production by NK cells [25, 26] and is secreted in response to LDV infection [27]. Therefore, we determined the requirement of this cytokine in the IFN-γ–dependent protection against mesothelioma growth in mice infected with LDV by in vivo neutralization with an IL-12 specific mAb. As shown in Fig. 5a, this treatment resulted in a significant decrease in the survival after tumor inoculation (*p* = 0.0337; representative of two independent experiments). Moreover, IFN-γ serum levels (Fig. 5b; *p* = 0.0317) and IFN-γ–producing NK cells (Fig. 5c; *p* = 0.0251) were significantly decreased after IL-12 neutralization. These results indicated that the protective effect of LDV infection on mesothelioma growth was largely mediated by an IL-12-dependent IFN-γ production by NK cells.

## Discussion

Modulation of the host immune microenvironment by infectious agents may lead to strong alterations of the course of concomitant diseases. The hygiene hypothesis suggests for example that the frequency of allergic and autoimmune diseases is rising in Western societies as a result of a decrease of common infections [28]. Animal models allow for the analysis of the mechanisms by which infections can modify the incidence of diseases not directly related to the pathogen, such as modulation of the immune system through interaction with innate receptors and cytokine production. LDV infection has been reported to decrease both allergic and autoimmune responses [29, 30] and provides therefore an interesting model to analyse the contribution of viral infections to this hygiene hypothesis. Could such an hygiene hypothesis apply to the development of cancers? Some reports showing an inverse relationship between an history of febrile infections and the risk to develop some cancers such as melanoma [31–33] may suggest so.

Although of poor prognosis, mesothelioma responds to immunotherapy both in patients and in animal models. In addition to T lymphocytes, NK cells have been reported to be involved in anti-mesothelioma response [15]. Using the classical AB1 mesothelioma mouse model, we found that an in vivo viral infection with LDV activates NK cells enough to prevent early mesothelioma development. Although T lymphocytes were clearly involved in the anti-mesothelioma response of normal animals, the role of NK cells could be observed in animals deprived of T lymphocytes, indicating that the mere activation of these NK cells was sufficient to provide at least some protection. This and other observations that experimental LDV infection reduces the development of other cancer types [11–13] through activation of the immune system rather than through direct oncolytic effect may thus support the hypothesis that modulation of the immune microenvironment by some pathogens leads to enhancement of immunosurveillance capacity of the infected host against some cancer types.

Our observation confirms also that NK cells play a major role in an efficient immunosurveillance against a range of tumors [1, 13, 14]. Distinct activation pathways are known to trigger different NK cell effector functions, including cytotoxicity and IFN-γ secretion. IL-2 for example is required to induce anti-mesothelioma activity by NK cells [15, 34]. Although other cytokines might also participate to this protective effect, we could observe involvement of IL-12, a classical NK cell activating cytokine, in LDV-induced protection, through modulation of IFN-γ secretion by these cells. This fits well with a previous report that anti-mesothelioma T lymphocyte response positively correlates with the production of this cytokine [35] and with the involvement of IFN-γ in LDV-mediated protection against plasmocytoma growth [13]. These observations confirm that IFN-γ is a major mediator of tumor immunosurveillance [3, 13, 36]. Therefore, it might be expected that any environmental mechanism resulting in enhanced IFN-γ secretion rather than in increased NK cell cytotoxic activity might contribute to protect against mesothelioma development. Other possible mechanisms that might be involved in enhanced cancer immunosurveillance include stimulations of innate receptors, like TLRs. This could be the case for LDV that has been shown to activate TLR-7 [9].

However, when installed, mesotheliomas develop a stong suppressive local immune microenvironment [37]. This correlates with impaired functional activity of NK cells [32, 36], suggesting that this tumor microenvironment inhibits their protective effect. Such a suppressive effect may explain why LDV infection does not protect any more when occuring after tumor cell inoculation. It may thus be postulated that infections that result in prolonged NK cell activation might be efficient in enhancing immunosurveillance of early cancer development, when just a few transformed cells are not yet surrounded by a local immunosuppressive microenvironment. In contrast, when cancer is installed, infection-triggered NK cell activation could no more stop cancer progression. Therefore, similarly to the hygiene hypothesis that suggests that a preventive effect on allergic and autoimmune responses results from constant exposure to various benign infectious agents, we may postulate that any protective effect of infections on cancer development would require a constant or frequent enhancement of cancer immunosurveillance through repeated exposure to infectious agents triggering activation of appropriate immune responses, including IFN-γ-producing NK cells.

## Conclusion

Our results extend, in an experimental model of solid tumor, evidence that viral infections can enhance cancer immunosurveillance, through activation of NK cells and enhanced production of IFN-γ. This suggests that a better understanding of the relationships between infections and cancer immunosurveillance in humans might provide useful information for a more efficient targeting of cancer early detection campaigns, especially in countries with scarse resources devoted to health programmes.

## Data Availability

The datasets used and/or analysed during the current study are available from the corresponding author on reasonable request.

## Abbreviations

NK: Natural Killer
IFN-γ: Gamma-interferon
IL: Interleukin
LDV: Lactate dehydrogenase-elevating virus
ASGM1: Asialoganglioside-GM1
mAb: Monoclonal antibody
PBS: Phosphate buffered saline
SEM: Standard error of mean
